# Visualization of an Accelerated Electrochemical Reaction under an Enhanced Electric Field

**DOI:** 10.34133/2021/1742919

**Published:** 2021-02-17

**Authors:** Chen Cui, Rong Jin, Dechen Jiang, Jianrong Zhang, Junjie Zhu

**Affiliations:** State Key Laboratory of Analytical Chemistry for Life Science, School of Chemistry and Chemical Engineering, Nanjing University, Nanjing 210023, China

## Abstract

Locally enhanced electric fields produced by high-curvature structures have been reported to boost the charge transport process and improve the relevant catalytic activity. However, no visual evidence has been achieved to support this new electrochemical mechanism. Here, accelerated electrochemiluminescence (ECL) reactions emitting light are visualized for the first time at the heterogeneous interfaces between microbowls and the supporting electrode surface. The simulation result shows that the electric intensity at the interface with a high curvature is 40-fold higher than that at the planar surface. Consequently, local high electric fields concentrate reactive species to the heterogeneous interfaces and efficiently promote the charge transport reactions, which directly leads to the enhancement of ECL emission surrounding the microbowls. Additionally, the potential to induce visual ECL from a ruthenium complex drops to 0.9 V, which further illustrates the promotion of an electrochemical reaction with the aid of an enhanced electric field. This important visualization of electric field boosted electrochemical reactions helps to establish the proposed electron transfer mechanism and provide an alternative strategy to improve electrocatalytic efficiency.

## 1. Introduction

As heterointerface structures play an important role in reduction of energetic barriers and acceleration of reaction kinetics for electrocatalytic system, a variety of optimal heterostructure materials have been synthesized to improve the performance of electrocatalysts [1–3]. Recently, many strategies have been proposed to fabricate interfaces in heterostructures, including doping of heteroatoms [4, 5], construction of heterostructures with different components [6, 7], and precise design of atomic metal-active sites [8–10]. These studies have suggested that heterogeneous interfaces exhibit particularly electrocatalytic activities because of their high-curvature morphologies, which is highly beneficial for concentrating electric fields that can affect the distribution of ion concentrations and accelerate charge transfer process [11–13]. Therefore, a low overpotential or amplified electrochemical currents would be achievable at these high-curvature sites.

Although many experimental results indicate that a locally enhanced electric field could accelerate an electrochemical reaction, visual evidence is needed to establish this novel electrochemical mechanism and directly image these electrochemical active sites. As an emerging electrochemical imaging approach, electrochemiluminescence microscopy has the ability to visualize an electrochemical reaction in situ at the electrode surface with a high spatial resolution [14–16]. When a certain potential is applied to the ECL system, the luminophores undergo electrochemical oxidation or reduction at the electrode surface, which motivates the formation of excited states that emit light [17–19]. Recently, the ECL collected by a charge-coupled device (CCD) has been suggested to be correlated with the electrochemical activity at the single particle level, indicating the high spatial resolution of ECL microscopy technique [20–22]. In contrast to scanning approaches using nanoprobes [23–25], ECL imaging is particularly suitable for visualizing electrochemical reactions at highly curved surfaces with heterogeneous electric fields. The image obtained could provide visual evidence to elucidate the proposed electrochemical mechanism.

**Scheme 1:**
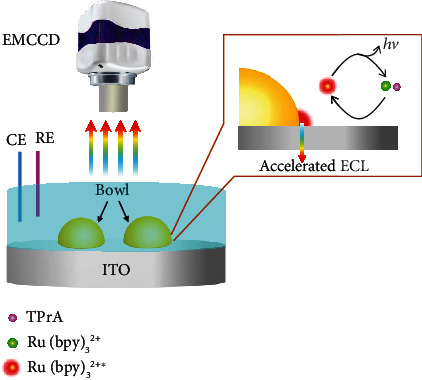
Schematic representation of the electrochemiluminescence imaging. The luminophore Ru(bpy)_3_^2+^ and coreactant TPrA are oxidized at the heterogeneous interface between the microbowls and the ITO supporting electrode with the aid of enhanced electric field, generating the excited state Ru(bpy)_3_^2+^^∗^. The accelerated ECL emission is produced during the relaxation of Ru(bpy)_3_^2+^^∗^ back to the ground state.

In this communication, a heterogeneous interface is designed by loading bowl-like microparticles (referred to as “microbowls”) onto the supporting indium tin oxide (ITO) electrode (Scheme 1), where an enhanced electric field is produced at the heterointerface between the microbowl and ITO electrode. The EM-CCD images a bright ECL ring surrounding the microbowl as a result of the accelerated electrochemical reaction from the luminophore at the heterointerface. The COMSOL simulations and experimental results confirm the existence of locally enhanced electric field, which is able to significantly promote the ECL emission.

## 2. Result and Discussion

### 2.1. Locally Enhanced Electric Field for Accelerating the Electrochemical Reaction

**Figure 1:**
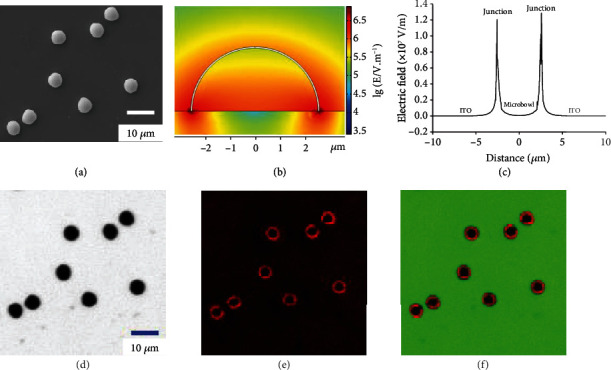
Computed electric field and ECL images at the heterogeneous interface. (a) SEM images of Au microbowls in a face-down configuration on the supporting surface. (b) The simulated electric field at the ITO surface with Au microbowls (face-up view). The rainbow bar is the logarithm value of the electric field intensity. (c) The electric strength at the heterogeneous interface with the microbowl and planar ITO surface. (d) Bright-field, (e) ECL, and (f) overlapping image of Au microbowls on the ITO slide. The ECL images are recorded in 10 mM PBS (pH = 7.2) that contains 5 mM Ru(bpy)_3_^2+^ and 50 mM TPrA.

These microbowls (e.g., Au and Pt) are prepared by using polystyrene (PS) particles as the template (Figure S1). Scanning electron microscopy (SEM) images (Figure S2) confirm that the Au hemispherical shell has a bowl-like structure and a thickness of ~60 nm. The outer diameter (OD) of these microbowls is controlled by the size of the PS particles (e.g., the OD is 5.0 ± 0.12 *μ*m using 5 *μ*m PS particles). After drop-casting these as-fabricated microbowls onto an ITO slide, over 80% of the microbowls in a face-down configuration make contact between the rim of the bowls and the supporting slide (Figure 1(a) and Figure S2d). The face-down configuration could provide a large contact area that stabilizes the microbowls on the ITO slide. According to the literature and our previous derivation [26, 27], an enhanced electric field is present at the interface between two mediums with different dielectric properties. Therefore, the electric field line around the heterointerface between the microbowl and ITO surface is more intense than that at the planar surface, which increases the electric strength. To support this statement, the potential drop is simulated using COMSOL software (see Experimental Section). A large potential drop adjacent to the heterointerface between the microbowl and ITO surface is obtained (Figure S4). According to the equation below, an increased potential gradient result in an increased electric intensity at the heterointerface (Figure 1(b)) [28, 29]:

(1) $$\begin{array}{c} E=-\nabla V, \end{array}$$

where *V* is the potential and *E* is the electric strength. The intensity at the heterointerface is ~40-fold larger than that at the planar surface 1.0 *μ*m away from the heterointerface (Figure 1(c)), confirming the proposed enhanced electric field at the heterointerface.

Experimentally, the ITO slide loaded with gold microbowls is exposed to 10 mM PBS (pH = 7.2) with 5 mM Ru(bpy)_3_^2+^ and 50 mM TPrA (Figure 1(d)). A periodic switching voltage (-1 V for 0.5 s and increased to +1.2 V for 2 s) is applied on the ITO electrode. Under the potential of 1.2 V, Ru(bpy)_3_^2+^ and TPrA are electrochemically oxidized at both the ITO slide and the microbowls emitting ECL. As expected, a visible ECL ring is observed surrounding the microbowl exhibiting an enhanced ECL intensity (Figure 1(e)). The image (Figure 1(f)) that overlaps the bright-field and ECL images confirms that accelerated ECL occurs at the heterointerface with a high electric field. The average ECL intensities at the heterointerface, ITO, and microbowl surface are measured to be 548.1, 128.7, and 177.8 a.u., respectively. The ECL intensity at the gold microbowl is higher than that at the ITO surface, owing to the superior electrocatalytic performance at gold material. In comparison with the ECL signals from the ITO and microbowl, an ~4.3-fold increase in ECL intensity at the heterointerface provides the first visual evidence of an accelerated ECL reaction under a locally enhanced electric field.

### 2.2. Simulation of the Electrochemical Reaction at the Junction

**Figure 2:**
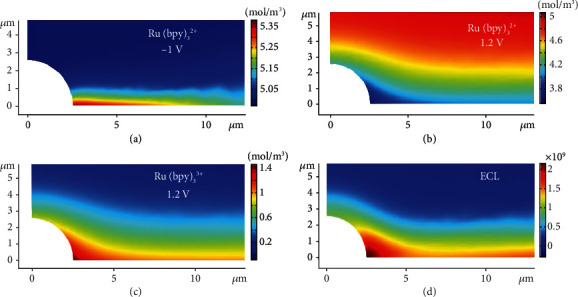
Simulated electrochemical reaction near the heterogeneous interface. (a) The simulated concentration distribution of Ru(bpy)_3_^2+^ before oxidation. The simulated concentration distribution of (b) Ru(bpy)_3_^2+^ and (c) Ru(bpy)_3_^3+^ after oxidation. (d) The simulated ECL intensity at the heterogeneous interface after oxidation.

To further understand this electric field accelerating electrochemical reaction, the concentration distributions from the Ru(bpy)_3_^2+^, Ru(bpy)_3_^3+^, and the ECL profiles at the heterointerfaces are simulated by COMSOL software (more detail in Experimental Section). All the charge transfer reactions and mass transport are considered comprehensively in the system. At an initial potential of -1 V, the concentration of Ru(bpy)_3_^2+^ is slightly higher than that at the planar electrode (Figure 2(a)), which should be ascribed to additional negative surface charges at the heterointerface with a high electric field. Following Pauling's principle of electroneutrality, electrons stored on the electrode surface from the external circuit are equal to the total number of excess charges in the diffuse layer [30, 31]. Therefore, additional Ru(bpy)_3_^2+^ should be present at the heterointerface, which is similar to the previously reported concentration effect under a high electric field [12]. When the potential is increased to 1.2 V, the oxidation of Ru(bpy)_3_^2+^ and TPrA generates ECL emission. The dynamic simulation displays the consumption of Ru(bpy)_3_^2+^ and the formation of Ru(bpy)_3_^3+^. In particular, less Ru(bpy)_3_^2+^ and more Ru(bpy)_3_^3+^ at the heterointerface are observed than those at the microbowl and ITO surfaces (Figures 2(b) and 2(c)). Moreover, enhanced ECL emission is obtained at the heterointerface from the simulation (Figure 2(d)). All these experimental and simulation results provide a possible mechanism for the acceleration of the ECL reaction. In summary, an enhanced electric field induces the initial accumulation of reactants and additional product generation during the electrochemical reaction, emitting a bright ECL light at the heterointerface.

### 2.3. Visualization of Accelerated Electrochemical Reaction under Different Conditions

**Figure 3:**
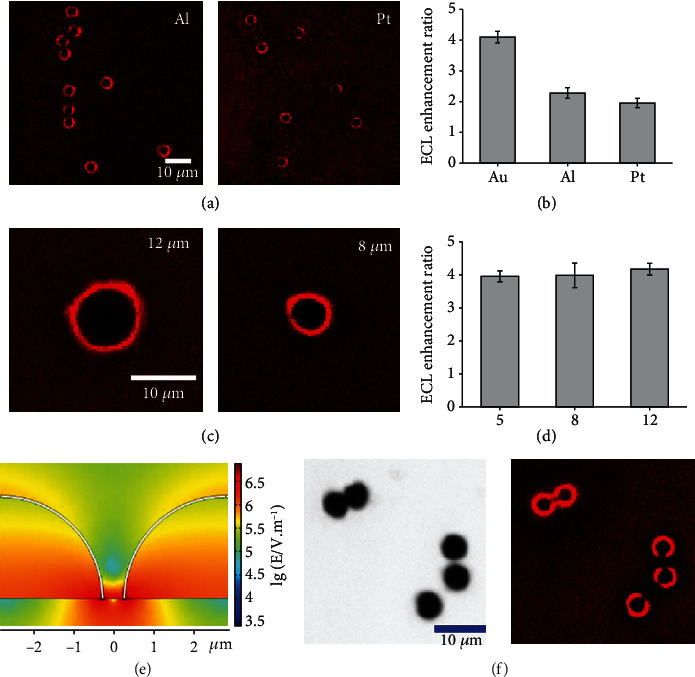
ECL images of different microbowls: (a) ECL images of Al and Pt microbowls loaded at the ITO surface; (b) the enhancement ratio of ECL intensity between the heterogeneous interface (with different microbowl materials) and the ITO surface; (c) ECL images of Au microbowls with the size of 12 and 8 *μ*m; (d) the enhancement ratio of ECL intensity between the heterogeneous interface (with different microbowl size) and the ITO surface; (e) the simulated electric field at the ITO surface with two adjacent microbowls; (f) the bright-field and ECL images from the adjacent microbowls. The ECL reagents are 5 mM Ru(bpy)_3_^2+^ and 50 mM TPrA in 10 mM PBS.

To confirm this visual evidence, the ITO electrode coated with Au microbowls is immersed into PBS solution containing another luminophore, luminol, to initiate the ECL reaction. An obvious blue ECL ring encircles the Au microbowls (Figure S5), whose intensity is ~2.45-fold stronger than that at the surfaces of both the ITO and microbowl. As compared with the enhancement ratio from Ru(bpy)_3_^2+^, the variance in the ECL enhancement from luminol is ascribed to the difference in the ECL efficiencies from these two luminophores. The observation of ECL rings from different luminophores illustrates that various ECL reactions could be accelerated at the heterointerface with an enhanced electric field. Subsequently, different materials (Pt and Al) are used to prepare the microbowls for ECL observation. Red ECL rings with an enhanced intensity are clearly observed around the Pt or Al microbowls in the Ru(bpy)_3_^2+^ and TPrA solutions (Figure 3(a)). The intensities of the ECL rings follow the trend of Au > Al > Pt, which is consistent with the trend of their conductivities (Figure 3(b)). Since a large discrepancy in the conductivity of the two materials at the interface leads to an enhancement in the electric field, the trend in the intensity of the ECL ring conforms to the theoretical expectations. Finally, Au microbowls 8 or 12 *μ*m in size are cast onto the surface of the ITO to study the size-dependent effect on the ECL enhancement (Figure 3(c)). A similarly enhanced ECL is observed from microbowls with different sizes (Figure 3(d)), which indicates that the similar curvature of the contact between the bowl and the supporting ITO might not change significantly the electric field intensity at the interface. Overall, although different microbowl materials and luminophores alter the enhancement ratio of the ECL at the heterointerface, the formation of an increased ECL at this region with a high electric field is clear, supporting the acceleration of an electrochemical reaction under a high electric field.

Interestingly, the location of two adjacent microbowls is found to create a low electric field region between these microbowls. In principle, as the two microbowls gradually approach each other, electrostatic repulsion from the electrons at the bowls occurs, resulting in electric fields with opposite directions. Thus, a weakened electric field should be formed between the microbowls in the vertical direction. To illustrate the distribution of the electric field, the spatial distribution of the local electric field and potential around the two microbowls is simulated (Figure 3(e)). Sparse equipotential lines between the two microbowls are clearly exhibited (Figure S6a). The electric field strength at 1.0 *μ*m away from the microbowl drops from 11 kV/cm (without an adjacent microbowl) to 0.31 kV/cm (with an adjacent microbowl) (Figure S6b). Correspondingly, the ECL image (Figure 3(f)) exhibits a defective ECL ring with a gap facing the adjacent microbowls, where a low electric field slows the ECL reaction between Ru(bpy)_3_^2+^ and TPrA. Although a shorter distance between these two microbowls could produce less electric field strength and the resultant slower electrochemical process, the diffusion of electroactive species is also present with a distance of 1~2 *μ*m that prohibits the decrease in ECL intensity. Consequently, a bright ECL ring with the darkest gap is observed with a distance of 1~2 *μ*m between two microbowls. The correlation of the electric field and the ECL intensity provides further visual evidence about the acceleration of an electrochemical reaction by a local electric field.

### 2.4. Initiate of Electrochemical Reaction at Low Potential

**Figure 4:**
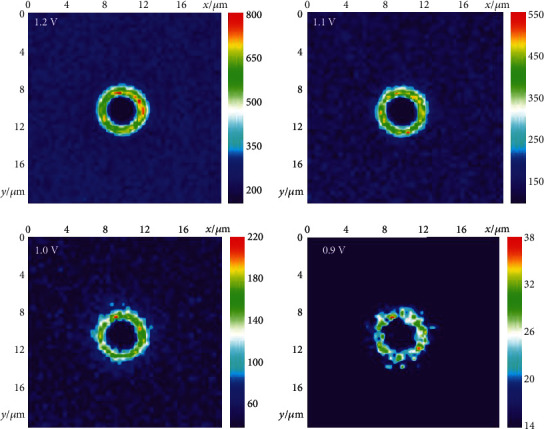
ECL images at different voltages. The false-color ECL images of microbowls at the supporting ITO surface applied with different voltages. The color presents the ECL intensity.

In electrochemistry, the achievement of a low potential to initiate an electrochemical reaction is important in many fields, such as electrical analysis and energy [32–34]. After validating the acceleration of an electrochemical reaction under an enhanced electric field, the potential to induce the reaction between Ru(bpy)_3_^2+^ and TPrA is attempted to decrease it from 1.2 to 0.9 V. The ECL images (Figure 4) show that the ECL intensities at the Au microbowl and ITO surface decrease with less potential and become almost invisible with a potential of 0.9 V. Some strong ECL spots observed at the ring should be ascribed to the presence of some sharp protrusions at the rim of microbowls (Figure S2d), which lead to the concentrated electric field and the resultant enhanced ECL emission. Excitingly, the ECL ring at the heterointerface is still visible under the potential of 0.9 V, which promotes an electrochemical reaction with the aid of an enhanced electric field. This important observation not only supports the acceleration of an electrochemical reaction by the enhanced electric field but also provides an alternative strategy to improve the electrochemical reaction efficiency.

## 3. Conclusions

In summary, we provide visual evidence to directly support a new electrochemical mechanism in which a locally enhanced electric field at an electrode surface could accelerate an electrochemical reaction. The heterogeneous distribution of the electric field could be realized by the design of materials with special structures at the supporting electrode. The establishment of this new mechanism sheds light on the crucial role of the electric field in electrocatalysis. In addition, some electrochemical reactions can be performed at low applied potential by the assistance of an enhanced electric field, promoting the practical application of electrochemistry in relevant fields. More microstructures with different shapes and sizes are being fabricated in the laboratory by changing the topography of the template. The study at these microstructures would solidify the conclusion about accelerated electrochemical reaction under enhanced electric field.

## 4. Experimental Section

### 4.1. Chemicals

Ru(bpy)_3_Cl_2_ and tripropylamine (TPrA) were purchased from Sigma-Aldrich. Colloidal suspension of monodisperse polystyrene (PS) microspheres with different diameters was purchased from Tianjin Beisile Chromatography Technology Development Center (Tianjin, China). Tetrahydrofuran solvent (Shanghai, China) was used to dissolve PS spheres. The sputtering targets (Au, Pt, Al ≥ 99.999%) were obtained from Zhongnuo Advanced Material (Beijing) Technology Corporation. ITO slides (coating thickness, 180 nm; resistance, <10 *Ω* m^−2^) were purchased from South China Xiangcheng Technology Co., Ltd. All other reagents were of analytical grade and were used without any purification. All aqueous solutions were prepared with ultrapure water obtained from a Milli-Q water purification system.

### 4.2. Fabrication of Microbowls

Experimental procedures to fabricate monodisperse Au microbowl particles are illustrated in Figure S1. Briefly, ordinary glass slides (8 × 15 mm) were ultrasonically cleaned in ultrapure water and ethanol solution, respectively. Then, the glass substrates were immersed into the piranha solution (concentrated H_2_SO_4_/30% H_2_O_2_, 3 : 1) to render the surface hydrophilic. Hydrophilic glass substrates were placed in a clean dish filled with 5 mL ultrapure water. All PS particles were dispersed in 1-butanol, which were dropped onto the water surface. Then, the monolayer colloidal film was formed on the water surface and was transferred to the glass slide by a peristaltic pump. Subsequently, the PS array particles on the glass slide were coated with gold conductive layer at a certain thickness using sputtering deposition. The gold-coated PS array on the glass slide was put into a 2 mL centrifuge tube filled with tetrahydrofuran solvent. The particles were detached from the glass slide after the sonication for 10 s, and the polystyrene templates were completely dissolved in tetrahydrofuran after 12 h. The microbowls with nanoshells were obtained by centrifugation at 8000 rpm for 10 min. Finally, the microbowls were dispersed with 1 mL of ethanol solution and maintained at 4°C.

### 4.3. Assembly of the Au Microbowl-Coated ITO Electrode

The purchased ITO electrode was cut into a 1.5 × 2.5 cm rectangular piece with a glass cutter, and these pieces were cleaned ultrasonically in acetone, ethanol, and ultrapure water, respectively. The cleaned ITO pieces were dried with nitrogen. Then, the solid-state PDMS O-ring with a diameter of 1.0 cm was prepared by hole punch, which was bonded to the conductive surface of ITO electrode with liquid PDMS. Subsequently, the ITO electrode-bonded PDMS O-rings were dried on a hot plate at 120°C for 2 min to form an electrochemical cell. A 10 *μ*L of ethanol containing ~4 × 10^4^ microbowl particles was added into the electrochemical cell. With the rapid evaporation of the ethanol solvent, the microbowls naturally fell on the surface of the ITO electrode. Finally, the ITO electrode covered with microbowls was dried on the hot plate for the ECL imaging.

### 4.4. ECL Imaging

The ECL imaging was performed with a three-electrode setup, consisting of a microbowl-coated ITO working electrode, an Ag/AgCl reference electrode (RE), and a Pt-counter electrode (CE). In order to measure ECL, a periodically switching voltage (-1 V for 0.5 s and stepped to 1.2 V for 2 s) was applied on the working electrode in 10 mM PBS (pH = 7.2) containing 5 mM Ru(bpy)_3_^2+^ and 50 mM TPrA. The exposure time was set to 2.5 s to collect ECL images, which were recorded by the optical microscopy coupled with a 40x objective and an EM-CCD camera.

### 4.5. COMSOL Multiphysics Simulations

The COMSOL Multiphysics 5.4 was used to simulate the distribution of electric field and equipotential lines in the junction between gold microbowls and the ITO surface. The electrostatics module was utilized to solve the electric field in the vicinity of the junction under a potential of 1.2 V. The electric field (*E*) was calculated as the opposite gradient of the electric potential V as follows: *E* = −∇*V* [12, 28]. The electric conductivity of the gold microbowls and the ITO electrode was taken to be 4.42 × 10^7^ S/m and 2.0 × 10^5^ S/m, respectively [35]. The electrolyte conductivity was assumed to be 10 S/m [36]. Gauss's law was used to compute charge density (*ρ*), according to the equation: *ρ* = *ε*_r_*ε*_0_∇·*E*, where *ε*_0_ and *ε*_r_ were the dielectric function for a vacuum and materials, respectively [12]. The *ε*_r_ equaled 78 for the electrolyte and 1 for gold.

In this work, to simulate the electrochemical reaction in the electrical double layer, an electrochemical reaction cell consisting of gold microbowl, supporting ITO electrode and electrolyte, was fabricated in electrical analysis module of COMSOL software. The boundary and domain settings are shown in Figure S3. The charge transfer reactions took place on the surface of the Au microbowl (domain 3) and supporting electrode surface (domain 1). The homogeneous chemical reactions occurred in the electrolyte (domain 2). In numerical simulation, charge transfer reaction and mass transport were considered in the electrochemical reaction. The mass transport was determined by the Nernst-Planck equation [37]:

(2) $$\begin{array}{c} \frac{\partial C_{i}}{\partial t}+\nabla J_{i}=R_{i},\\ J_{i}=-D_{i}\nabla C_{i}-\frac{Z_{i}F}{RT}D_{i}C_{i}\nabla \varphi . \end{array}$$

The oxidation and reduction of Ru(bpy)_3_^2+/3+^ redox pair follow the Butler-Volmer equation [38].

(3) $$\begin{array}{c} i_{\text{loc}}=Fk_{0}\left\{C_{\text{red}}\exp\left(\frac{1-\alpha F}{RT}\Delta \varphi \right)-C_{\text{ox}}\exp\left(\frac{-\alpha F}{RT}\Delta \varphi \right)\right\}. \end{array}$$

Based on the Butler-Volmer equation, current density *i*_loc_ was the function of heterogeneous reaction rate *k*_0_, concentration of oxidized/reduced species, and overpotential Δ*φ*. Transfer coefficient *α* was taken as 0.5. The heterogeneous reaction rate *k*_0_ of the ITO electrode and gold microbowl was taken to be 5 × 10^−4^ m/s and 1.59 × 10^−5^ m/s, respectively. To simulate the concentration distribution of the Ru(bpy)_3_^2+/3+^ redox pair at the junction between gold microbowl and ITO electrode, a time-dependent study on transport of diluted species was considered. All reaction equations in Table S1 and parameters in Table S2 were from previous reports [39–41]. According to variables c1–c8 in Table S3, the homogenous and heterogeneous reactions of ECL were modulated in the 2D model fabricated by the COMSOL software.

## Data Availability

All data needed to support the findings of this study are present in the paper and the Supplementary Materials.

## Supplementary Materials

Supplementary Materials Table S1: reactions involving the ECL process. All the reactions are from the previous reference. P in represents the product of TPrA after losing electrons and protons. Table S2: parameters for digital simulation. All the parameters are from the previous report. Table S3: variables used in digital simulation. The equations in the table represent the reaction speed of the substance. Figure S1: schematic illustration for the fabrication of monodisperse gold microbowls. The PS microsphere (with a diameter of 5 *μ*m) arrays were formed on the glass substrates by using the assembly method. The gold layer was deposited on the PS arrays with the aid of sputtering equipment. The monodisperse gold microbowls were obtained via the sonication and removing PS template. Figure S2: gold microbowl SEM and EDS images. SEM images of (a) face-up and (b) face-down microbowls. (c) SEM-EDS elemental map of Au microbowls. (d) SEM image of microbowls at ITO slide demonstrating most face-down microbowls. Figure S3: model of electrochemical reaction cell. The boundary (a) and domain (b) number settings for the whole electrochemical reaction interfaces. Figure S4: simulated spatial distribution of electric field lines and equipotential lines. The COMSOL software was used to simulate the distribution of electric field lines (white lines) and equipotential lines (rainbow lines) near a gold microbowl with inner diameter of ~5 *μ*m. The electric field lines were perpendicular to the equipotential lines, and the white arrow indicated the direction of the electric field lines. Figure S5: ECL imaging of ~5 *μ*m gold microbowls in the luminol solution. Bright-field image (a) and ECL image (b) of single gold microbowl at ITO slide recorded in the 0.5 mM luminol solution in 10 mM PBS (pH 7.4). The potential was held at -1.0 V for 0.5 s and 1.0 V for 2 s. Scale bar: 10 *μ*m. Figure S6: electric field distribution of two adjacent gold microbowls. (a) The simulated spatial distribution of electric field lines (white lines) and equipotential lines (rainbow lines) located at ITO electrode with two adjacent gold microbowls. (b) The computed electric strength at the heterogeneous interface with two adjacent microbowls and planar ITO surface.

## References

[1] Zheng X., Han X., Cao Y. (2020). Identifying dense NiSe_2_/CoSe_2_ heterointerfaces coupled with surface high-valence bimetallic sites for synergistically enhanced oxygen electrocatalysis. *Advanced Materials*.

[2] Ji D., Sun J., Tian L. (2020). Engineering of the heterointerface of porous carbon nanofiber–supported nickel and manganese oxide nanoparticle for highly efficient bifunctional oxygen catalysis. *Advanced Functional Materials*.

[3] Yang G., Jiao Y., Yan H. (2020). Interfacial engineering of MoO_2_-FeP heterojunction for highly efficient hydrogen evolution coupled with biomass electrooxidation. *Advanced Materials*.

[4] Singh S. K., Takeyasu K., Nakamura J. (2019). Active sites and mechanism of oxygen reduction reaction electrocatalysis on nitrogen-doped carbon materials. *Advanced Materials*.

[5] Wang T., Yang R., Shi N. (2019). Cu,N-codoped carbon nanodisks with biomimic stomata-like interconnected hierarchical porous topology as efficient electrocatalyst for oxygen reduction reaction. *Small*.

[6] Yang Y., Wang Y., He H.-L. (2020). Covalently connected Nb_4_N_5_–xOx–MoS_2_ heterocatalysts with desired electron density to boost hydrogen evolution. *ACS Nano*.

[7] Diao J., Qiu Y., Liu S. (2020). Interfacial engineering of W_2_N/WC heterostructures derived from solid-state synthesis: a highly efficient trifunctional electrocatalyst for ORR, OER, and HER. *Advanced Materials*.

[8] Su X., Yang X.-F., Huang Y., Liu B., Zhang T. (2019). Single-atom catalysis toward efficient CO_2_ conversion to CO and formate products. *Accounts of Chemical Research*.

[9] Fei H., Dong J., Chen D. (2019). Single atom electrocatalysts supported on graphene or graphene-like carbons. *Chemical Society Reviews*.

[10] Pan F., Li B., Sarnello E. (2020). Atomically dispersed iron–nitrogen sites on hierarchically mesoporous carbon nanotube and graphene nanoribbon networks for CO_2_ reduction. *ACS Nano*.

[11] Sage A. T., Besant J. D., Lam B., Sargent E. H., Kelley S. O. (2014). Ultrasensitive electrochemical biomolecular detection using nanostructured microelectrodes. *Accounts of Chemical Research*.

[12] Liu M., Pang Y., Zhang B. (2016). Enhanced electrocatalytic CO_2_ reduction via field- induced reagent concentration. *Nature*.

[13] De Luna P., Mahshid S. S., Das J. (2017). High-curvature nanostructuring enhances probe display for biomolecular detection. *Nano Letters*.

[14] Ding H., Guo W., Su B. (2020). Imaging cell-matrix adhesions and collective migration of living cells by electrochemiluminescence microscopy. *Angewandte Chemie International Edition*.

[15] Zhang J., Jin R., Jiang D., Chen H.-Y. (2019). Electrochemiluminescence-based capacitance microscopy for label-free imaging of antigens on the cellular plasma membrane. *Journal of the American Chemical Society*.

[16] Voci S., Goudeau B., Valenti G. (2018). Surface-confined electrochemiluminescence microscopy of cell membranes. *Journal of the American Chemical Society*.

[17] Voci S., Duwald R., Grass S. (2020). Self-enhanced multicolor electrochemiluminescence by competitive electron-transfer processes. *Chemical Science*.

[18] Yu L., Zhang Q., Kang Q., Zhang B., Shen D., Zou G. (2020). Near-infrared electrochemiluminescence immunoassay with biocompatible Au nanoclusters as tags. *Analytical Chemistry*.

[19] Chen J., Wang Q., Liu X., Chen X., Wang L., Yang W. (2020). Black phosphorus quantum dots as novel electrogenerated chemiluminescence emitters for the detection of Cu^2+^. *Chemical Communications*.

[20] Chen M.-M., Zhao W., Zhu M.-J. (2019). Spatiotemporal imaging of electrocatalytic activity on single 2D gold nanoplatesviaelectrogenerated chemiluminescence microscopy. *Chemical Science*.

[21] Ma C., Wei H.-F., Wang M.-X. (2020). Hydrogen evolution reaction monitored by electrochemiluminescence blinking at single-nanoparticle level. *Nano Letters*.

[22] Guo W., Ding H., Zhou P., Wang Y., Su B. (2020). Electrochemiluminescence waveguide in single crystalline molecular wires. *Angewandte Chemie International Edition*.

[23] Sun T., Wang D., Mirkin M. V. (2019). Direct high-resolution mapping of electrocatalytic activity of semi-two-dimensional catalysts with single-edge sensitivity. *Proceedings of the National Academy of Sciences of the United States of America*.

[24] Polcari D., Dauphin-Ducharme P., Mauzeroll J. (2016). Scanning electrochemical microscopy: a comprehensive review of experimental parameters from 1989 to 2015. *Chemical Reviews*.

[25] Mahankali K., Thangavel N. K., Reddy Arava L. M. (2019). In situ electrochemical mapping of lithium–sulfur battery interfaces using AFM–SECM. *Nano Letters*.

[26] Jin R., Huang Y., Cheng L., Lu H., Jiang D., Chen H.-Y. (2020). *In situ* observation of heterogeneous charge distribution at the electrode unraveling the mechanism of electric field-enhanced electrochemical activity. *Chemical Science*.

[27] Huang Z.-F., Song J., Du Y. (2019). Optimizing interfacial electronic coupling with metal oxide to activate inert polyaniline for superior electrocatalytic hydrogen generation. *Carbon Energy*.

[28] Saberi Safaei T., Mepham A., Zheng X. (2016). High-density nanosharp microstructures enable efficient CO_2_ electroreduction. *Nano Letters*.

[29] Kim S., Dong W. J., Gim S. (2017). Shape-controlled bismuth nanoflakes as highly selective catalysts for electrochemical carbon dioxide reduction to formate. *Nano Energy*.

[30] Forse A. C., Merlet C., Griffin J. M., Grey C. P. (2016). New perspectives on the charging mechanisms of supercapacitors. *Journal of the American Chemical Society*.

[31] Lian C., Liu K., Liu H., Wu J. (2017). Impurity effects on charging mechanism and energy storage of nanoporous supercapacitors. *The Journal of Physical Chemistry C*.

[32] Kumatani A., Miura C., Kuramochi H. (2019). Chemical dopants on edge of holey graphene accelerate electrochemical hydrogen evolution reaction. *Advanced Science*.

[33] Li Y., Zhang L. A., Qin Y. (2018). Crystallinity dependence of ruthenium nanocatalyst toward hydrogen evolution reaction. *ACS Catalysis*.

[34] Wang Y., Wang S., Li R. (2020). A simple strategy for tridoped porous carbon nanosheet as superior electrocatalyst for bifunctional oxygen reduction and hydrogen evolution reactions. *Carbon*.

[35] Ban S.-G., Kim K.-T., Choi B. D. (2017). Low-temperature postfunctionalization of highly conductive oxide thin-films toward solution-based large-scale electronics. *ACS Applied Materials & Interfaces*.

[36] Hu Y.-F., Zhang X.-M., Li J.-G., Liang Q.-Q. (2008). Semi-ideal solution theory. 2. Extension to conductivity of mixed electrolyte solutions. *The Journal of Physical Chemistry B*.

[37] He R., Chen S., Yang F., Wu B. (2006). Dynamic diffuse double-layer model for the electrochemistry of nanometer-sized electrodes. *The Journal of Physical Chemistry B*.

[38] Zevenbergen M. A. G., Wolfrum B. L., Goluch E. D., Singh P. S., Lemay S. G. (2009). Fast electron-transfer kinetics probed in nanofluidic channels. *Journal of the American Chemical Society*.

[39] Miao W., Choi J.-P., Bard A. J. (2002). Electrogenerated chemiluminescence 69: the tris(2,2‘-bipyridine)ruthenium(II), (Ru(bpy)_3_^2+^)/tri-n-propylamine (TPrA) system revisited a new route involving ^TPrA•+^ cation radicals. *Journal of the American Chemical Society*.

[40] Ma C., Wu W., Li L. (2018). Dynamically imaging collision electrochemistry of single electrochemiluminescence nano-emitters. *Chemical Science*.

[41] Zhu M.-J., Pan J.-B., Wu Z.-Q. (2018). Electrogenerated chemiluminescence imaging of electrocatalysis at a single Au-Pt Janus nanoparticle. *Angewandte Chemie International Edition*.

