# Supplementary material for: Visualization of an Accelerated Electrochemical Reaction under an Enhanced Electric Field
Source: Research (Wash D C). 2021 Feb 17;2021:1742919. doi: 10.34133/2021/1742919 (PMC7907821; doi:10.34133/2021/1742919)
Supplement: Supplementary Materials — Table S1: reactions involving the ECL process. All the reactions are from the previous reference. P in represents the product of TPrA after losing electrons and protons. Table S2: parameters for digital simulation. All the parameters are from the previous report. Table S3: variables used in digital simulation. The equations in the table represent the reaction speed of the substance. Figure S1: schematic illustration for the fabrication of monodisperse gold microbowls. The PS microsphere (with a diameter of 5 μm) arrays were formed on the glass substrates by using the assembly method. The gold layer was deposited on the PS arrays with the aid of sputtering equipment. The monodisperse gold microbowls were obtained via the sonication and removing PS template. Figure S2: gold microbowl SEM and EDS images. SEM images of (a) face-up and (b) face-down microbowls. (c) SEM-EDS elemental map of Au microbowls. (d) SEM image of microbowls at ITO slide demonstrating most face-down microbowls. Figure S3: model of electrochemical reaction cell. The boundary (a) and domain (b) number settings for the whole electrochemical reaction interfaces. Figure S4: simulated spatial distribution of electric field lines and equipotential lines. The COMSOL software was used to simulate the distribution of electric field lines (white lines) and equipotential lines (rainbow lines) near a gold microbowl with inner diameter of ~5 μm. The electric field lines were perpendicular to the equipotential lines, and the white arrow indicated the direction of the electric field lines. Figure S5: ECL imaging of ~5 μm gold microbowls in the luminol solution. Bright-field image (a) and ECL image (b) of single gold microbowl at ITO slide recorded in the 0.5 mM luminol solution in 10 mM PBS (pH 7.4). The potential was held at -1.0 V for 0.5 s and 1.0 V for 2 s. Scale bar: 10 μm. Figure S6: electric field distribution of two adjacent gold microbowls. (a) The simulated spatial distribution of electric field lines ( [file 1742919.f1.docx]

*Supplementary Information*

**Visualization of an accelerated electrochemical reaction under an enhanced electric field**

**Chen Cui, Rong Jin, Dechen Jiang,* Jianrong Zhang,* Jun-Jie Zhu***

*State Key Laboratory of Analytical Chemistry for Life Science, School of Chemistry and Chemical Engineering, Nanjing University, Nanjing 210023, China.*

**Table S1：**Reactions involving the ECL process. All the reactions are from the previous reference [1]. P in the table S1 represents the product of TPrA after losing electrons and protons.

| Charge transfer reactions | Ru(bpy)_3_^2+^ - e → Ru(bpy)_3_^3+^ | (1) |
| --- | --- | --- |
|  | TPrA - e → TPrA**^·+^** | (2) |
|  | TPrA**^·^** - e → P | (3) |
| Homogeneous reactions | H_2_PO_4_^-^ → H^+^+ HPO_4_^2-^ | (4) |
|  | TPrA + H_2_PO_4_^-^ → TPrAH^+^ + HPO_4_^2-^ | (5) |
|  | TPrA**^·+^** → TPrA**^·^** + H^+^ | (6) |
|  | Ru(bpy)_3_^3+^ + TPrA**^·^**→ Ru(bpy)_3_^2+*^ + P | (7) |
|  | Ru(bpy)_3_^2+^ + TPrA**^·^** → Ru(bpy)_3_^+^ + P | (8) |
|  | Ru(bpy)_3_^3+^ + Ru(bpy)_3_^+^ → Ru(bpy)_3_^2+*^ + Ru(bpy)_3_^2+^ | (9) |
|  | Ru(bpy)_3_^3+^ + TPrA → Ru(bpy)_3_^2+^ + TPrA**^·+^** | (10) |
|  | Ru(bpy)_3_^+^ + TPrA**^·+^** → Ru(bpy)_3_^2+*^ + TPrA | (11) |
|  | Ru(bpy)_3_^2+*^ → Ru(bpy)_3_^2+^ + *hv* | (12) |

**Table S2：**Paremeters for digitial simulation. All the paremeters are from the previous report [2,3].

| Category | Name | Value | Description |
| --- | --- | --- | --- |
| Initial concentration | c_0-Ru2_ | 5 mol/m^3^ | Initial concentration of Ru(bpy)_3_^2+^ |
|  | c_TPrA0_ | 50 mol/m^3^ | Initial concentration of TPrA |
|  | c_TPrAH0_ | 50 mol/m^3^ | Initial concentration of TPrAH |
|  | c_PBS0_ | 10 mol/m^3^ | Initial concentration of PBS |
|  | c_H0_ | 6.3096 × 10^-5^ mol/m^3^ | Initial concentration of H^+^ |
| Diffusion coefficient | D_Ru_ | 1 × 10^-9^ m^2^/s | Diffusion coefficient of Ru(bpy)_3_^2+^, Ru(bpy)_3_^3+^ and Ru(bpy)_3_^+^ |
|  | D_TPrA_ | 5 × 10^-10^ m^2^/s | Diffusion coefficient of TPrA, TPrA**^·+^** and TPrA**^·^** |
|  | D_H_ | 9.3 × 10^-9^ m^2^/s | Diffusion coefficient of H^+^ |
|  | D_Buf_ | 5 × 10^-10^ m^2^/s | Diffusion coefficient of PBS |
| Reactions rate | k_1_ | 537 1/s | Rate constant for TPrA**^·^** generation |
|  | k_4_ | 1 × 10^7^ m^3^/(s.mol) | Rate constant for Ru(bpy)_3_^+^ generation |
|  | k_5_ | 1 × 10^7^ m^3^/(s.mol) | Rate constant for Ru(bpy)_3_^2+*^ generation |
|  | k_6_ | 1 × 10^-3^ m^3^/(s.mol) | Rate constant for TPrA**^·+^** consumption |
|  | k_7_ | 1 × 10^7^ m^3^/(s.mol) | Rate constant of Ru(bpy)_3_^2+^ reaction |
|  | k_8_ | 1 × 10^3^ m^3^/(s.mol) | Rate constant of Ru(bpy)_3_^+^ reaction |
|  | k_9_ | 15848 1/s | Rate constant for ECL generation |
|  | k_b-Au_ | 1.59 × 10^-5^ m/s | Reaction rate constant of Ru(bpy)_3_^2+^ on Au microbowls surface |
|  | ks_2_ | 0.1 m/s | Rate constant for TPrA**^·+^** generation on ITO surface |
|  | ks4 | 0.1 m/s | Rate constant for P generation on ITO surface |
|  | ke | 5 × 10^-4^ m/s | Reaction rate constant of Ru(bpy)_3_^2+^ on ITO surface |
| Particle sizes | i-bead | 5 × 10^-6^ m | The inner diameter of Au microbowls |
|  | o-bead | 5.12 × 10^-6^ m | The outer diameter of Au microbowls |
|  | Dapp | 1 × 10^-13^ m^2^/s | Boundary diffusion coefficient of Ru(bpy)_3_^2+^ |

**Table S3：**Variables used in digital simulation. The equations in the table represent the reaction speed of the substance.

| Name | Expression | Equation |
| --- | --- | --- |
| Ru(bpy)_3_^+^ | c1 | -k1 × c8 + k5 × c2 × c7 |
| Ru(bpy)_3_^2+^ | c2 | k9 × c4 |
| Ru(bpy)_3_^3+^ | c3 | -k4 × c3 × c7 |
| Ru(bpy)_3_^2+*^ | c4 | k4 ×c3 × c7 - k9 × c4 |
| hv | c5 | k9 × c4 |
| TPrA | c6 | k8 ×c1 × c8 |
| TPrA**^·^** | c7 | k1 × c8 |
| TPrA**^·+^** | c8 | -k1 × c8 |

**Figure S1：**Schematic illustration for the fabrication of monodisperse gold microbowls. The PS microsphere (with a diameter of 5 μm) arrays were formed on the glass substrates by using the assembly method. The gold layer was deposited on the PS arrays with the aid of sputtering equipment. The monodisperse gold microbowls were obtained via the sonication and removing PS template.

**Figure S2：**Gold microbowl SEM and EDS images. SEM images of (a) face-up and (b) face-down microbowls. (c) SEM-EDS elemental map of Au microbowls. (d) SEM image of microbowls at ITO slide demonstrating most face-down microbowls.


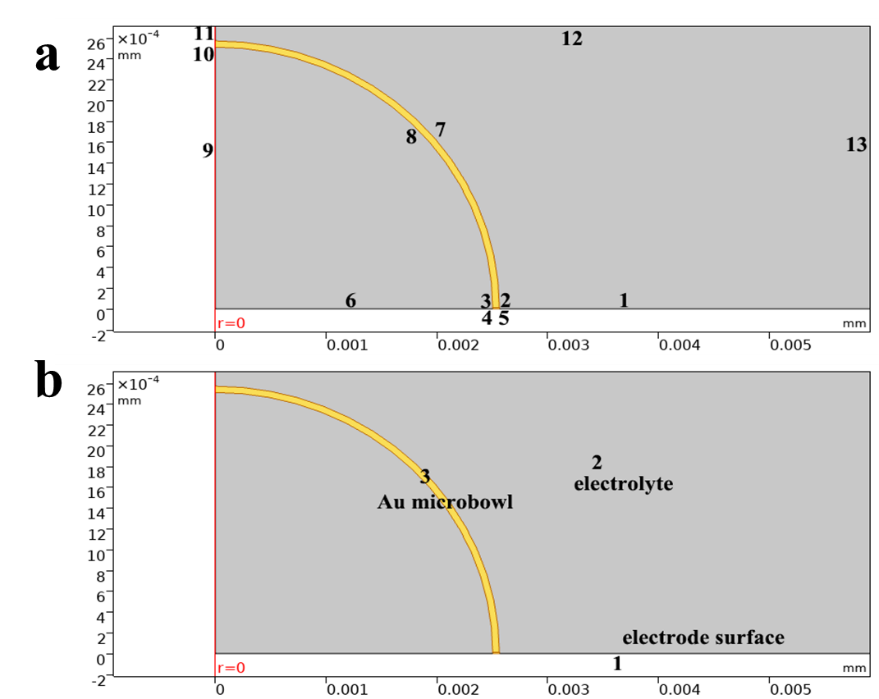


**Figure S3：**Model of electrochemical reaction cell. The boundary (a) and domain (b) number settings for the whole electrochemical reaction interfaces.


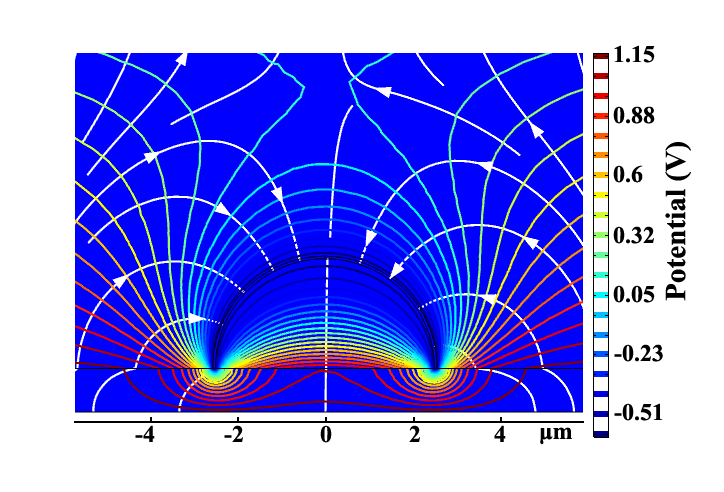


**Figure S4:** Simulated spatial distribution of electric field lines and equipotential lines. The COMSOL software was used to simulate the distribution of electric field lines (white lines) and equipotential lines (rainbow lines) near a gold microbowl with inner diameter of ~5 μm. The electric field lines were perpendicular to the equipotential lines and the white arrow indicated the direction of the electric field lines.


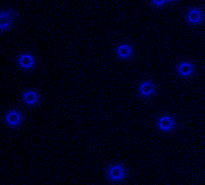

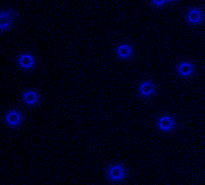

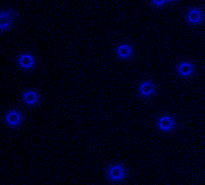


**b**


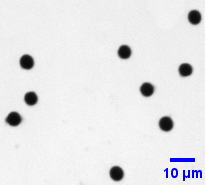


**a**


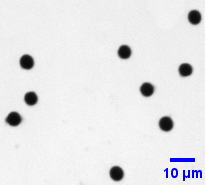


**Figure S5:** ECL imaging of ~5 μm gold microbowls in the luminol solution. Bright field image (a) and ECL image (b) of single gold microbowl at ITO slide recorded in the 0.5 mM luminol solution in 10 mM PBS (pH 7.4). The potential was held at -1.0 V for 0.5 s and 1.0 V for 2 s. Scale bar: 10 μm.


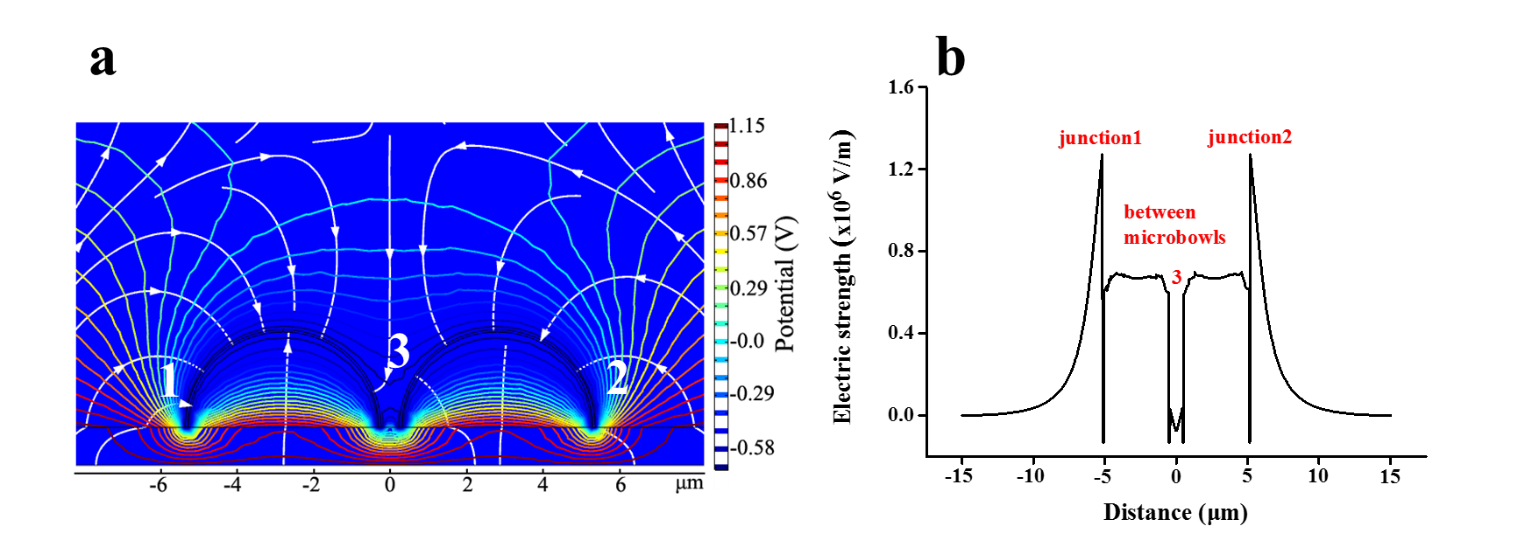


**Figure S6:** Electric field distribution of two adjacent gold microbowls. (a) The simulated spatial distribution of electric field lines (white lines) and equipotential lines (rainbow lines) locating at ITO electrode with two adjacent gold microbowls. (b) The computed electric strength at the heterogeneous interface with two adjacent microbowls and planar ITO surface.

**References**

1. W. Miao, J.-P. Choi, and A. J. Bard, “Electrogenerated Chemiluminescence 69:  The Tris(2,2‘-bipyridine)ruthenium(II), (Ru(bpy)_3_^2+^)/Tri-n-propylamine (TPrA) System RevisitedA New Route Involving TPrA^•+^ Cation Radicals,” *Journal of the American Chemical Society*, vol. 124, no. 48, pp. 14478-14485, 2002.
2. C. Ma, W. Wu, L. Li et al., “Dynamically imaging collision electrochemistry of single electrochemiluminescence nano-emitters,” *Chemical Science*, vol. 9, no. 29, pp. 6167-6175, 2018.
3. M.-J. Zhu, J.-B. Pan, Z.-Q. Wu et al., “Electrogenerated Chemiluminescence Imaging of Electrocatalysis at a Single Au-Pt Janus Nanoparticle,” *Angewandte Chemie International Edition*, vol. 57, no. 15, pp. 4010-4014, 2018.
